# Structural Health Monitoring (SHM) and Determination of Surface Defects in Large Metallic Structures using Ultrasonic Guided Waves

**DOI:** 10.3390/s18113958

**Published:** 2018-11-15

**Authors:** Muntazir Abbas, Mahmood Shafiee

**Affiliations:** Department of Energy and Power, Cranfield University, Bedfordshire MK43 0AL, UK; m.shafiee@cranfield.ac.uk

**Keywords:** structural health monitoring (SHM), guided waves ultrasonic testing (GWUT), metallic structures, non-destructive evaluation (NDE), ultrasonic guided wave (UGW), remaining useful life (RUL)

## Abstract

Ultrasonic guided wave (UGW) is one of the most commonly used technologies for non-destructive evaluation (NDE) and structural health monitoring (SHM) of structural components. Because of its excellent long-range diagnostic capability, this method is effective in detecting cracks, material loss, and fatigue-based defects in isotropic and anisotropic structures. The shape and orientation of structural defects are critical parameters during the investigation of crack propagation, assessment of damage severity, and prediction of remaining useful life (RUL) of structures. These parameters become even more important in cases where the crack intensity is associated with the safety of men, environment, and material, such as ship’s hull, aero-structures, rail tracks and subsea pipelines. This paper reviews the research literature on UGWs and their application in defect diagnosis and health monitoring of metallic structures. It has been observed that no significant research work has been convened to identify the shape and orientation of defects in plate-like structures. We also propose an experimental research work assisted by numerical simulations to investigate the response of UGWs upon interaction with cracks in different shapes and orientations. A framework for an empirical model may be considered to determine these structural flaws.

## 1. Introduction

Mechanical structures and their reliability deteriorate continuously under the influence of operating and ambient environmental conditions. The structural degradation processes may occur in different forms of corrosion, fatigue cracks, erosion, and strength reduction [1]. The extent and severity of these mechanisms depend on the applied static/dynamic loads, operating conditions, and material properties such as corrosion resistance, microstructural features, the crystallographic orientation and the grain-boundary structure [2]. The inspection and maintenance of mechanical structures were performed mainly in accordance with a preventive (time-based) approach in the past. In recent years, the significance of structural health monitoring (SHM) techniques has been enhanced in the health assessment, remaining useful life (RUL) prediction, and condition-based maintenance (CBM) of degrading structures [3].

The SHM approach involves collecting data from various sensors installed on structures and interpreting the findings to make reliable and cost-effective and CBM decisions for structures under different operating conditions. Numerous health assessment techniques such as bulk wave ultrasonic, X-rays, infrared thermography, and eddy current are used effectively for non-destructive evaluation (NDE) of structures. However, majority of these techniques tend to be slow and cumbersome, especially in case of large structures (pipelines, marine, ships, and aerospace). The guided waves ultrasonic testing (GWUT) potentially offers a smart alternative solution to conventional approach towards NDE and SHM [4]. The guided wave (GW) has a unique ability to confine itself inside the thin-wall structures, therefore, they can propagate over large distances with minimal attenuation and loss of energy [5]. They are also known as long-range ultrasonic testing (LRU), introduced initially to determine the integrity assessment of large and continuous pipelines [6,7]. The GWs have the potential to be used inside curved structures thus suitable for inspection of various shapes and geometries over a longer distance. 

**Figure 1:**
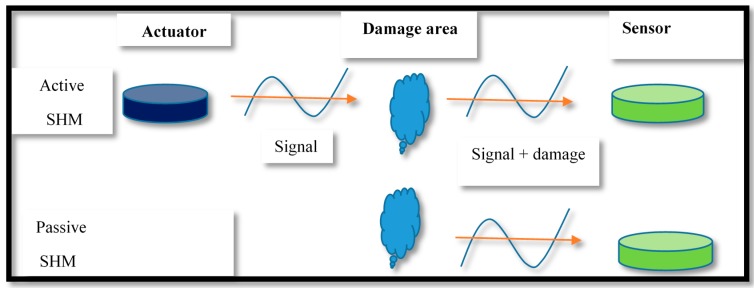
Active and passive SHM methods.

The SHM is generally performed by two methods: the passive and active SHM [8]. The passive SHM is an examining method used in static/dynamic and rotating equipment. It requires external excitation source to produce elastic waves in structures and utilize the functioning parameters such as vibration and stress levels for health assessment. However, the passive SHM does not provide the root cause(s) of the problem and the opportunity for a thorough examination of the defect. Contrary, the active SHM is a direct health assessment method for detection and estimation of structural defects using health detection act structures actuators/sensors. Like NDE, the SHM sensors detect and quantify the structural defect but in the latter technique sensors are permanently fixed/embedded in structures; to provide health monitoring data on need basis [8,9,10]. A schematic view of active and passive SHM methods is shown in Figure 1.

The UGW is a rapidly emerging SHM technique for inspection of metallic/composite structures [11,12]. The prominent features of GWUT such as high sensitivity to surface/subsurface flaws, long reach, efficacy in finding faults of inaccessible areas have made this approach a cost-effective and preferable choice for structural health assessment [8,10,13]. Contrary to the conventional ultrasonic testing system which offers defect diagnosis only under immediate vicinity of a diagnostic probe, the GWs (Lamb waves in particular) are able to identify defects at larger distances by virtue of its distinct low frequencies and tendency to confine with the structural surfaces [14]. It has the potential to examine various types of structural flaws such as gouges, deep corrosion spots, cracks and notches using one or more sensors [3,15,16,17]. GWs have been used to detect, localized the defects and corrosion under insulation (CUI) in pipelines and various metallic/non-metallic surfaces [18,19]. 

This paper aims to review research publications on structural defect detection with GW technique, particularly in metallic structures. It signifies the achievements of this technique to detect various features of defects (crack/notches) on metal surfaces. We identify certain research gaps with respect to the classification of some crucial facets (shape and orientation of cracks/notches) of structural flaws, detectable by UGW techniques. Furthermore, to the best of our knowledge, the determination of these characteristics of defects (using UGW method) in plate type structures has not been addressed in any research study to date. To overcome the research gap, this paper also proposes an experimental research assisted by numerical simulation on thin metal plates (aluminium). It aims to determine the signal variations in excited Lamb waves (short tone burst) after interacting with the surface cracks (through-thickness and partial through-thickness) in several shapes and orientations. Based on the theoretical knowledge and previously held research work on GWs, it seems quite possible to sense the projected changes in an output signal (amplitude, reflection/transmitted coefficients, acoustic impedance and arrival time) upon interaction with these aspects of structural discontinuities.

The rest of the paper is organized as follows: Section 2 describes the types, specifications, and salient facets of guide waves for SHM. Section 3 presents the literature review, deliberating the scope, capability, application areas, and implications of this technology. Section 4 comprises discussion of the results, proposes an experimental plan to apply UGWs, especially in SHM of large marine structures. Section 5 concludes the study and discusses directions for future research.

## 2. Ultrasonic Guided Waves and SHM

The GWs are elastic waves transmitting in form of wave packets, and confined along the structures with distinct boundaries. The propagation of GWs depends on the material properties and slight variation may take place with variation in environmental conditions. These waves are highly dispersive, and almost infinite modes are generated by the superposition of basic longitudinal and transverse waves. The Lamb wave is one of the main types of GWs used in SHM. They are elastic perturbations normally traveling with a different phase (C_p_) and group (C_g_) velocity in metals. In here, the displacement of medium occurs both in the direction of wave propagation as well as in a perpendicular direction [20]. The Shear and Rayleigh waves are the other types of GWs which are also used in SHM and NDE. These waves travel with slower speed than the Lamb waves [10]. The vibration of Lamb waves may be observed through the entire thickness of the medium.

The GWs are able to travel over long distances without any significant loss of energy [21]. Although certain complications are also associated with the GW technology in SHM such as multimode excitation, dispersive behavior, boundary scattering, and difficulty in interpretation [22]. Nevertheless, several approaches (e.g., variation in incident pulse frequency, pulse shape, short tone burst, filters, signal processing method) have been used to minimize the undesirable features of GWs [23,24]. 

When the elastic waves are propagated on a metallic surface, the induced stress waves tend to produce elastic strain [25,26]. The damage/discontinuities in structures cause exclusive wave scattering and mode conversions, which can be visualized on the output sensor. The SHM techniques can be used to capture these crack transitions in the form of variation in reflected/transmitted waves, which can be further utilized for identification of vicinity of defect and its quantification [27]. Contrary to the labour intensive, time-consuming, and expensive inspections by other techniques, GWUT can be helpful to inspect wider structural area in less time, cost and cheap equipment setup [28,29].

The UGWs have been used in health prognostic studies in several ways including contact method, semi contact, and non-contact probing techniques [30]. In the contact method, various types of acoustic sensors are used which are either bonded (with epoxy) or embedded in structural surfaces. The excitation and sensing of acoustic waves can be achieved by contact transducers of several types, normally made of an electro-active and magneto-active substance. These substances have the ability to change their features when induced by an electric or magnetic signature and respond in the form of strain actuation/sensing. The piezoelectric polymers (PVDF), piezoelectric ceramics (PZT), electrostrictive ceramics (PMN), and magnetostrictive materials (Terfenol-D) are the types of transducer used in SHM. 

The electromagnetic acoustic transducers (EMATs) are a non-contact technique based on the principles of Lorentz force or magnetostriction which have been used enormously in defect diagnosis studies. The EMATs are ideal to directly excite and detected S_H_ waves in the structures, however they are quite larger in size and their utility is restricted to the conductive materials only [31,32,33]. The piezo wafer active sensor (PWAS) and S_H_-PWAS have also been used for damage detection [34]. These acoustic transducers can be utilized in various arrangements like wedge, square, circular, comb and phase array type [8,10,35,36]. The PZT is more frequently used for SHM in aerospace, mechanical and civil engineering appliances. It works on the electro-mechanical (EM) impedance/admittance principle. The EM admittance signatures can be used for structural health assessment and flaw detection corresponding to the signal variations [37]. PWAS can be used in several ways such as frequency response transfer function, E/M impedance and wave propagation [8]. A new type of a thin, lightweight and flexible transducers is Shear-horizontal piezoelectric fiber patch (SH-PFP), which has been adopted for exciting the S_H_ waves in light structures in aerospace [32,33,38] 

The propagation of GWs can be divided into two broad categories: longitudinal and transverse waves. When these waves propagate in a traction-free metal plate, the longitudinal wave particles tend to travel along the direction of wave propagation (in from of compressions and rarefactions) whereas the transverse wave particles oscillate perpendicular to the direction of wave propagation. The velocity of acoustic waves depends on various properties of the medium of propagation, as shown in the following equations:

(1) $$C_{T}=\sqrt{\frac{E}{2\rho \left(1+V\right)}}$$

(2) $$C_{L}=\sqrt{\frac{E\left(1-V\right)}{\rho \left(1-2V\right)\left(1+V\right)}}$$

where the term *E* is Young’s modulus, V is Poisson’s ratio, ρ is density, and C_T_ and C_L_ represent transverse and longitudinal wave velocities, respectively. Readers can also refer to [8,10,25,26,39,40,41] for a detailed study on the characteristics and application of long-range UGWs in metallic and composite structures.

### 2.1. Lamb Guided Waves

The Lamb ultrasonic guide waves, also known as plate waves, are guided between two parallel traction free surfaces, such as the lower and upper surface of a plate. The Lamb wave speed is also dependent on the frequency of propagating waves [42]. The Lamb waves are a complicated form of GW molded by superimposition of longitudinal and Shear/transverse waves [40]. The two basic modes of Lamb waves are symmetric and antisymmetric modes. Both modes of Lamb waves are dispersive in nature and equally used for SHM. The former mode of waves travel faster while being affected less by the external conditions. The frequency of GW and thickness of structural are deeply correlated to define various features of the GWs. The frequency-depth (f-d) product is proportional to the number of Lamb-wave modes. 

**Figure 2:**
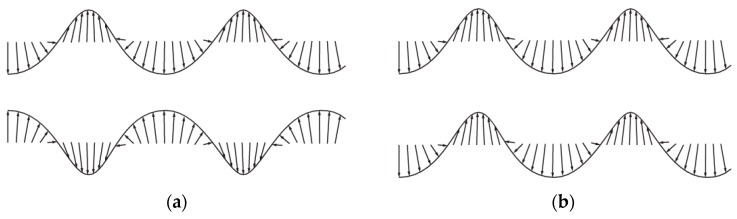
Schematic of (**a**) symmetric particle displacement (**b**) antisymmetric particle displacement (adapted from [10])*.*

Similarly, for smaller f-d product, only fundamental Lamb wave modes of symmetric and antisymmetric (i.e., S_0_ and A_0_) exist [8,36,43]. The shape and mode of propagation of particle in both symmetric and antisymmetric Lamb waves are illustrated in Figure 2. The use of antisymmetric modes of Lamb waves has been reported as a preventive technique for marine fouling deposition on underwater ship structure. In the structural plates immersed in liquid medium, the adequate selection of wave mode can significantly decrease energy leakage into the water [10,44].

Use of Lamb waves on structural defect detection was first started in early 1960. Since then, rigorous research studies have been conducted for application of these waves in health prognostics of metallic/composite structures [27,45,46,47,48]. Various SHM based studies have been conducted on Lamb waves behavior on metallic and non-metallic structural defects [27,45,47,49]. These waves have the greater tendency to propagate larger distances (up to 100 m) with little attenuation [50]. The real-time data of Lamb waves is also amalgamated with the external excitations, operational and environmental noises, further complicating the interpretation process analysis by shifting of the arrival time [37,51,52]. Therefore, various compensatory approaches have been introduced to minimize the signal damages. Chan and Cawley [53] studied the impact of attenuation on the dispersion behavior of Lamb wave in plastic plates (high-density polyethylene). The attenuation in Lamb wave was found to be five times less that of the Shear wave.

### 2.2. Shear Guided Waves 

**Figure 3:**
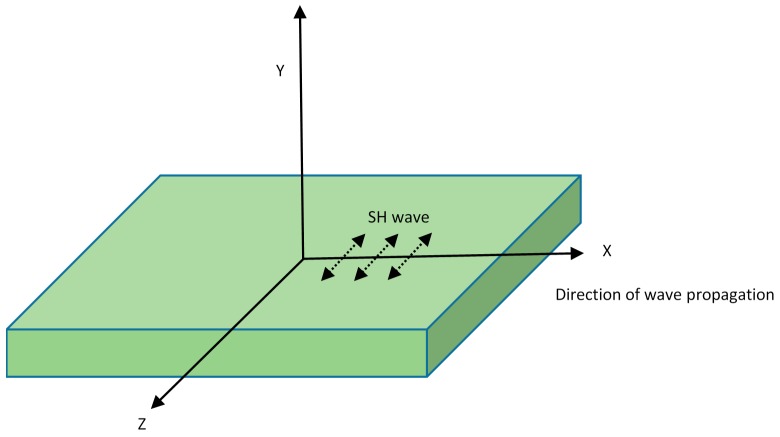
Shear wave propagation (adapted from [40]).

The Shear waves are also called transverse waves in which most of the movement of the medium is perpendicular to the direction of wave propagation. They exist in two forms: the Shear horizontal (S_H_) and the Shear vertical (S_V_) waves. The particle vibration of S_H_ waves is polarized parallel to the plate surface, as illustrated in Figure 3. The S_H_ waves exist in both symmetric and antisymmetric modes and both are dispersive in nature, with the exception of very fundamental S_H0_ mode [8]. The significance of S_H_ waves has increased in recent years by the inculcation of new techniques to overcome difficulties associated with S_H_ wave generation. Therefore, superior transducers have been introduced for using shear waves in SHM, such as magnetostrictive transducers and EMATs.

The S_H_ wave is used in ice detection and de-icing applications in various structures. They have also been preferred for NDT and health monitoring studies of underwater structures because they do not leak out from structural into the liquid medium. Therefore, the maximum propagation energy retains inside the submerged structure [10]. The S_H_ waves have the unique ability to propagate around curved surfaces with minimal energy loss. These waves can propagate circumferentially around the pipe structures [54]. The S_H_ waves are polarized in a plane parallel to a surface boundary so they can be reflected without substantial mode conversion and variation in amplitude. Likewise, S_H_ waves have less reflection, beam steering, and attenuation compared with the S_V_ waves; therefore they are considered as a preferred choice in defect detection in welded regions [55]. However, it has been observed that any sudden change in the surface thickness may lead to the mode conversion. 

### 2.3. Rayleigh Waves

**Figure 4:**
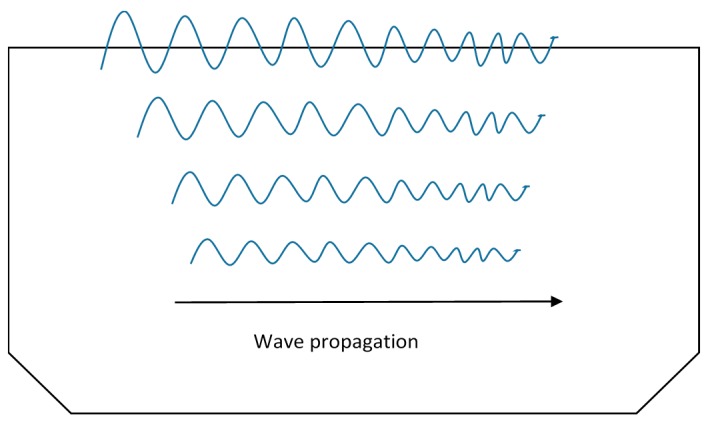
Rayleigh waves propagation (adapted from [10]).

Rayleigh waves are also known as surface acoustic waves (SAW) and normally found in solids having free surfaces. As S_V_ wave exists only along the thickness of the plate, it superposes with the longitudinal wave and forms a new type of wave called the Rayleigh wave. These waves travel close to the free surface with slight penetration in the depth. For this reason, Rayleigh waves are also known as surface guided waves [8]. The Rayleigh waves are polarized in a plane normal to the surface and wave particles propagate in a way, making the path reversing ellipses in the vertical plane. These waves occur on the surface of semi-infinite elastic media and suitable for surface and subsurface flaw detection [36]. Figure 4 shows Rayleigh wave propagation in metal plates. 

These waves travel over the surface of a semi-infinite solid or a very thick plate, where the wavelength is very small as compared to the thickness of the plate. These waves have also been used in NDE of the structural defects, confined to surfaces [56]. They can be used for inspection of artillery shells, acoustic microscopy, and leaky Lamb wave method for composite materials [3]. 

### 2.4. Phase and Group Velocity

The phase velocity (C_p_) of an acoustic wave is the rate at which the phase of the wave propagates in spatial dimensions. The individual harmonics travel with different C_p_, but the superimposed packet travels with the group velocity (C_g_). In dispersive elastic wave propagation, the packets of waves travel with a different velocity (C_g)_ than individual wave velocity C_p_ [8,10]. 

**Figure 5:**
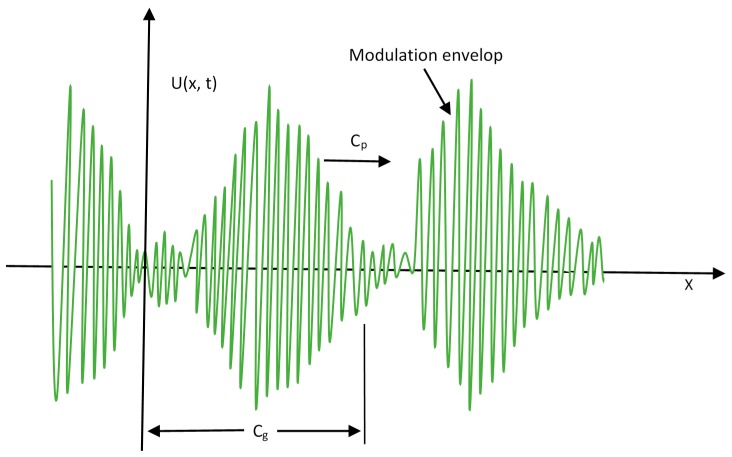
Phase and group velocities of GWs.

It has been revealed by Wilcox et al. [18] that the dispersion of GWs cause the wave-packets to disperse in space and time domains as they travel in a structure. The equations for C_p_ and C_g_ are given by:

(3) $$C_{p}=\left(\frac{\omega }{k}\right)$$

(4) $$C_{g}=\frac{\partial \omega }{\partial k}$$

where k is wave number and ω is angular velocity of waves. Figure 5 illustrates the phase and group velocities of GWs propagating in metal structures. During the propagation of ultrasonic GWs, the phase velocity is normally a function of the wave frequency and thickness of the structure under examination, therefore, it is obvious that for a given frequency, different thickness samples have different wave velocity [54].

### 2.5. Attenuation in Ultrasonic Guided Waves 

The combined effect of scattering and absorption is called attenuation. In ultrasonic GWs, the attenuation is the rate of decay of the waves as they propagate through any material. The energy of GWs dissipates gradually with distance which manifests the steady drop in magnitude of wave signals. Attenuation in UGWs has been reported as a function of wave modes, physical properties of the material, and frequency. The presence of any structural damage or inhomogeneity such as welds, joints, stiffeners or fasteners increases the dissipation factor [40]. As compared to other forms of GWs, the Lamb wave and S_H_ wave can travel longer distances with minimal loss in amplitude [55,57]. The application of surface coatings, intricacies in structural geometry and immersion/embedded conditions often result in high attenuation of acoustic waves [10]. These factors restrict the range of GWs and consequently degrade its quality of the defect identification [58]. The low excitation frequency often results in a phase-lead and substantial attenuation of the signal [59]. During a research investigation on phase velocity and attenuation of GWs in viscoelasticity of human tissues, Nenadic et al. [60] reported that the attenuation of the Shear wave was found to be 20% lower than that of Lamb–Rayleigh waves.

### 2.6. Sensor Placement Methods for GWUT 

The pitch-catch and pulse-echo are the most commonly used methods in defect detection. In a pitch-catch, GWs are excited by the actuator and only the waves transmitted across the damage region are captured/monitored by the sensor placed opposite to the exciter, in a catching position. This method normally uses one or more sensors. Any change in signal intensity/energy, velocity shift, arrival time, amplitude, and impedance can signify the characteristics of the damaged area [20].

**Figure 6:**
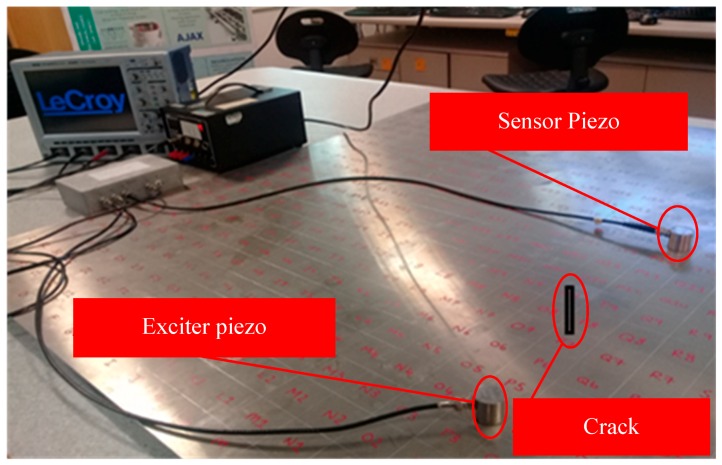
Pitch-catch method.

**Figure 7:**
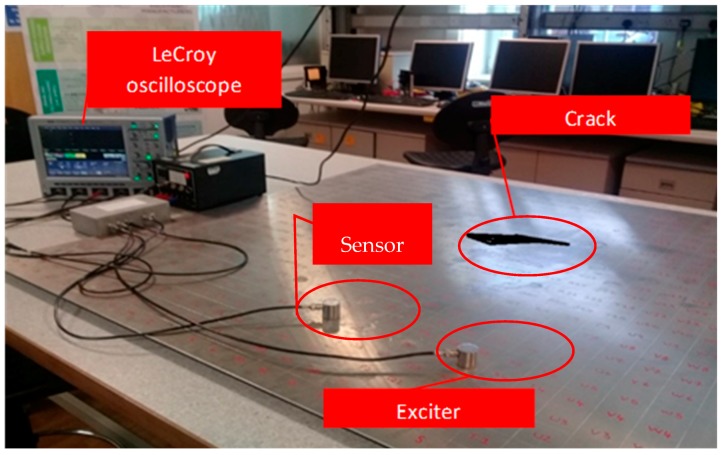
Pulse-echo method.

In pulse-echo, both the exciter and receptor sensors are located on the same side and the sensor receives the echoed wave (i.e., reflected part of the signal only) signal from any structural discontinuities or boundaries [61]. Figure 6 and Figure 7 exemplify both arrangements. In the pulse-echo method, the defect is detected in the form of additional echoes, whereas in the pitch-catch method, the principles of wave dispersion and attenuation are used as the defect indicators [8]. The pitch-catch configuration is also known as through transmission. Because of the wide coverage capability of the pitch-catch method, it has been proposed by few researchers for inspecting the floor of fuel storage tanks, using lamb waves [62,63].

The output signal received on the sensor is the exclusive source of information about the nature of the defect. It may encompass several changes in the characteristics of the induced signal after interaction with structural discontinuities. The error signal is obtained by taking the difference between undamaged and damaged signal. The highly advanced oscilloscopes (as shown in Figure 6) have the capability to carry out the basic system validation using advanced triggers, fast viewing modes, measurement parameters, or serial decodes. Additionally, the multi-domain analysis such as Fast Fourier transformation (FFT) and short-time Fourier transform (STFT) convert useful signal information from time domain into the easily interpretable frequency domain. These vigorous signal transfiguring functions are incorporated in these oscilloscopes (such as Lecroy wave-runners^®^ Xi series) for ease of signal processing, analysis, and interpretation.

## 3. Research Literature Review on Various Aspects of UGWs Technology

Over the years, the acoustic waves in form of ultrasonic, acoustic emission (AE), laser vibrometry, and UGWs have been used immensely for SHM and NDE of metallic and composite structures [49,57,64,65,66,67]. Apart from their applications in health prognostic studies, the GWs have been used for various other functions such as ice detection, de-icing, pigging, and antifouling operations in various isotropic and anisotropic structures [12,44]. Although, under some specific circumstances, the reach and efficacy of GWs is confined by the contribution of environmental factors such as temperature, moisture and humidity, and external excitations of running machinery [68,69,70].

The GWs scatter at various angles after interaction with structural defects and this scattering phenomena is proportional to the severity/size of discontinuity. It is difficult to collect most of the dispersed signals by limited number of sensors. Therefore, the use of multiple detached transducers in various array configurations has become instrumental to investigate large structural areas with better accuracy [71]. When a guided ultrasonic wave is stimulated with a crack, the excited input signal transforms and subsequently results in a difference of signal amplitude and phase shift between signal response of damaged and undamaged structures [72]. The sensitivity of GWs towards different defects in the thickness of the structure depends on the type and location of the damage as well [73]. The structural discontinuities and damage in mechanical structures can be examined by quantifying the various changes in output signal, including E/M impedance signatures [40,74]. The precise collection of the time of flight (TOF) has been considered as an important step to localize the damaged region in a structure. In an experimental study, Dai and He [75] revealed that the performance of a time–frequency method called Wigner–Ville Distribution (WVD) was found superior to the other signal transforming methods (Hilbert envelope and Gabor wavelet transform) in estimating the precise TOF of excited signal based on energy distribution. 

The GWUs have shown enormous potential in SHM of aviation based metallic and composite structures. These structures are prone to abrupt variation in ambient conditions of humidity level, ambient temperature, stress loading, and pressure that can instigate the corrosion and fatigue based structural cracks [51]. The traditional SHM techniques are labour- intensive and require considerable de-striping of assemblies and removal of surface protections for inspection. The frequent de-striping of machinery components may yield maintenance induced damages (MIDs) [76]. Therefore, the need for a more efficient, reliable, and optimized health monitoring system like GWUT is always a desirable element for operators and maintenance crew so that they can inspect a relatively large area with minimal disruption [77].

The ice deposition on structures may compromise the efficacy of NDE and remote monitoring systems by warning for a false damage or missing the detection of actual defect failure. More recently, some selective modes of guided waves have been used successfully for detection of ice on various metallic/composite structures such as wind turbine blades, aero-structures, helicopter rotors and ship’s hull. The icing phenomena is a critical issue in the lube oil/fuel circuits of aircrafts and their aerodynamic efficiency and maneuverability. Some researchers used GW modes to differentiate water and ice content as well as deicing on structures in cold climates. The guided waves produced by a piezoelectric transducer (bonded/embedded on a surface) can be used to induce shear stresses to delaminate/fracture ice deposits on various structural surfaces [78,79]. Displacement controlling of in-plane/out-of-plane wave modes excited on the outer surface of structure can be helpful to detect water or ice layers. 

In an ice sensing experiment, Zhao and Rose [80] deliberated the behaviour of S_0_ mode of Lamb wave to differentiate between the water and ice layers on aluminium plate (1.6-mm thick). A circular array of 16 sensors were used with an exciting frequency of 350 KHz. The S_0_ mode having dominant in-plane displacement on metal surface was found to be insensitive to the water layer. However, it remained very sensitive towards the ice loading. Later, the probability-based reconstruction (PRA) algorithm was used to construct the ice images in other types of material such as titanium and carbon-fiber-reinforced titanium plates. In case, ships maneuvering in the polar region, the amalgamated ice layers and water disturbances may cause variation in the signal response of GWs and the ice detection may be misleading the damage detection process. Memmolo et al. [81] conducted a numerical based study (finite element analysis with ABAQUS^®^) for ice detection on ship structures using both pitch-catch and pulse-echo methods. It was revealed that the back-scattering in the pulse-echo signal and forward scattering in the pitch-catch configuration are highly dependent on the length of the ice layer. This study proposed that it is quite much possible to quantify the dimensions of the ice layer.

In the cold countryside, icing of wind turbine structures (especially blades) is a common problem, which may hinder its operation. The ice layers on the surface of the blades can change the propagation characteristics of the acoustic waves used for condition monitoring. Wang et al. [82] investigated icing and temperature effect on wind turbine blades under extremely cold climates by a simulation carried out in a frozen tunnel. Three parameters, including amplitude, group velocity, and spectrum value of Lamb wave were used as indicators. The amplitude and spectrum of S_0_ wave mode were found to be more sensitive to the variations in the thickness of the ice loading than the temperature factor, whereas the group velocity of S_0_ mode was deeply influenced by the changes in both the temperature and the thickness of ice layer. Complexities in the estimation of ice formation and temperature variation were addressed effectively using a principal component analysis (PCA) based ice monitoring method.

During a research study on the use of antisymmetric (flexural) cylindrical GWs in the underwater pipelines, Na and Kundu [17] used a new transducer coupler and found it to be effective in identifying the presence of defects in butt welds, anti-corrosion covers, insulation and modest portions of soil/concrete embedded structures. However, some limitations were observed when allowed to pass through flanges and long embedded pipes. In the health monitoring of pipelines, the detection and quantification of wall thinning process is mainly focused which is caused by the internal or external corrosion. The external corrosion is generally found in areas where water is entrapped, which damages surface coatings, wall thinning and pitting corrosion [83]. The sensitivity of GWs towards corrosion defect would be different than that of a crack damage. The reflected part of the signal generated in case of corrosion defect will be comparatively smaller but the TOF shift would be different depending upon the severity of the corrosion loss and variation in the group velocity of the propagated wave. The research study of Gao and Rose [84] revealed that the variation in TOF is a function of corroded section on the structure. The reflected and transmitted coefficients of GW signal has been used to detect accumulations of sludge content in pipelines [85].

Apart from use of GWUT in structures, Tse and Rostami [86] demonstrated the application of this technique to defect assessment in wire rope cross-section, using magnetostrictive UGWs. The output signals were investigated using a Short Time Fourier Transform (STFT) as well as Wavelet analysis, and the experimental results were able to locate and identify the severity of imperfections. For the ease of signal interpretation, modulated pulses of GWs with short tone burst are generally preferred in experimental studies [49]. Some of these modulations such as rectangular and square modulated signal may not be feasible as they can hamper the signal processing by excitation of the larger spectrum and additional modes. The Gaussian-modulated waves have been preferred for signal excitation by some researchers [24,87]. Cawley and Alleyne [57] reported that in order to generate a pure mode of propagation, it is essential to regulate the bandwidth of excitation in both the frequency and wavenumber domains, which can be best achieved by using a short tone burst of Gaussian or Hanning window, in case of frequency. A limited cycle sinusoidal tone burst reduced undesired reflections between energy packets [51].

Time-shift average algorithms were used by Michaels and Michaels [88] in differential filtered signals to acquire numerous images of the same structure. These images were then merged to enhance the resolution of defect localization and signal to noise ratio (SNR). The increasing distance along the plate, however, results in rapid reduction of SNR. The structural cuts and impact damage on the trailing edge of the aluminium slat were investigated by Senyurek [27] using Hilbert transform. It was revealed that the cuts created new boundaries and the reflected waves were found to be helpful in estimating the location of the defects. The intricacy of reflected or transmitted signals may be moderated by using digital bandpass filtering hence any variation in filtered signals can be examined to identify and localize damages area of structures [88].

The advancement of sensor equipment over the years has benefitted SHM/NDE techniques and assisted in the optimization of maintenance/RUL prediction for structures [89]. The importance of CBM has enhanced in the modern-day maintenance due to developments in sensor technology. Conventional preventive, proactive and reliability-based maintenance strategies have been revamped with the addition of health monitoring data in overall decision-making for future maintenance actions and inspection intervals [90]. The GWUT can be an extremely useful addition in the CBM for structural health inspection and overall maintenance planning of the mega-structures. The variations in amplitude, and travel time of GWs have been used by various researchers for identification and location of structural cracks in aging structures and found it as an effective method for structural integrity inspections the [91,92]. The study by Alleyne and Cawley [93] revealed that the elastic propagation of Lamb waves on surface cracks of the metallic plate is reliant on the wave mode (symmetric or antisymmetric), its mode order, frequency-depth (f-d) product and the crack geometry. In a similar research study, Hall et al. [94] reported that the interaction of GWs with a surface defect is different from a sub-surface defect and it is primarily dependent on the defect size, angle of incidence, and excitation frequency.

Yu et al. [95] used UGWs for quantification of cracks in aluminium structures using an advanced wave field analysis methods. The A_0_ mode exhibited strong out of phase motion than S_0_ mode. It was noticed that the transmission index was found stronger than the reflection index in half-through-thickness cracks than full through-thickness cracks. This effect was attributed to the fact that the metal portion underneath the half-thickness crack allows transmission of forwarding wave. The material impedance is another factor for deciding transmission and reflection of the acoustic signal. The effect of defect orientation (metal surfaces) on reflection and transmission coefficients of GWs has been described in [40]. It has been revealed that the reflection coefficient reduces whereas the transmission coefficient rises with an increase in the angle (θ) of the incidence wave with respect to notch, hence depicting a strong angular dependence of the scattered Lamb waves. Conversely, with the same angle θ, the transmission coefficient declines with the notch length, while the reflection coefficient increases up to 60 mm of the notch length. Zima and Rucka [19] conducted experiment-based research assisted by numerical simulation for quantification of various sizes of cracks on a steel plate. A sine modulated Hanning widow was used for excitation of Lamb wave and a binary damage imaging algorithm was applied to identify the linearly crafted cracks with a greater accuracy. 

The GWUT has been able to diagnose cracks or CUIs in the bends/elbows of pipelines. Only a limited portion of the wrapped material is required to be removed for placement of sensors and defect diagnosis process is conducted without removal of entire material [7,96,97]. The axial cracks in carbon steel pipelines were examined by Qi et al. [98] using Shear wave. A good agreement is reported between experimental work and finite-element modeling (FEM) analyses. Highest detection sensitivity with fewer mode conversions was recorded in the middle area of elbow than in the intrados and extrados regions. The flexural mode of guided waves have been preferred for use in pipeline inspections, as the scattering pattern of these waves can be vital to differentiate between circular holes, cracks located at different orientations and slanting angles [99]. The authors of this paper believe that the research of Zhang et al. [100] can be extended for identification of shape and orientation of structural defects in thin plate type structures using Lamb GWs. In this regard, the research studies of Yu et al. [95] and Su and Ye [40] provided good grounds for identification of structural defects placed at various angles, shapes, and orientations in plate-like metallic structures.

**Figure 8:**
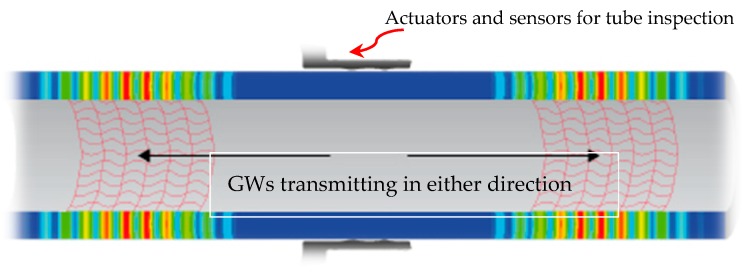
GWs inspection of a metallic pipeline structures [96].

In the oil and gas industry, the underground and subsea pipelines are more prone to general corrosion, fatigue, stress cracking and pitting [101]. Most of the times, these huge pipe networks carry petroleum fluids at high pressure/temperature and are exposed to extreme environmental sea conditions from the external side [102,103]. Apart from piping material properties, the degradation of these marine based structures depends on various physical, chemical and biological compositions of seawater [104]. Generally, flaw detection of underground or subsea pipes is carried out by in-line inspection (ILI) tools, also referred to as smart or intelligent pigs, working on the principles of magnetic flux leakage (MFL) or conventional ultrasonic inspection technique. Various limitations are involved in the use of these methods. The former technique has limitations to discriminate among various severity levels of defect and their position (external or internal surface). The latter technique can be extremely cumbersome and expensive in large pipe networks and difficult positions. Therefore, use of GWs has been proposed by Ángela et al. [105] for the pigging system, which has more reliable, longer area coverage with limit sensors, permanent and highly efficient. The standard practice ASTM E2775 [106] and Clough et al. [54] have also supported the plan to use GWUT for screening of defects in pipelines. Figure 8 depicts GWs propagation in metallic pipes.

In a non-contact approach (laser-based), a pulsed laser is used to generate elastic waves and a laser Doppler vibrometer or interferometers are used for collection and detection of structural discontinuities [107]. The Fabry-Pérot and heterodyne interferometers are often used for contactless acquisition of guided waves [40]. More recently, various defect-imaging techniques have been developed and the characteristics of low-frequency GWs have been used to obtain images of defects. The non-contact methods can also be used in both plate type and tubular structures. Defect imaging of the thinning process on the aluminium pipeline has been described in [108]. The high-power fiber-laser and a laser Doppler vibrometer (LDV) were used for generation and detection of flexural elastic waves (modulated frequency 22 kHz to 36 kHz). A semi-analytical finite element method (SAFEM) was used for phase velocity dispersion curves and the frequency image averaging (FIA) to minimize the undesirable circumferential resonance patterns. A fast imaging method (E-camera) was used for imaging of metal loss at all positions of a straight and branched pipes located at a distance of 2.6–6 m from E-camera.

In comparison with non-contact methods, some researchers believe that the contact based techniques have some limitations as follows [109]:

- Guided wave excitation and sensing is limited (by contact method) to some discrete points, therefore it is hard to achieve a high resolution to sense minor emerging defects;
- High cost for transducer, assemblies, installation, and labour cost. The vulnerability for equipment damage is high, leading to high maintenance budgets;
- Limitation for use of contact transducers under harsh environmental conditions and application to convoluted/high speed rotating surfaces such as high temperature/pressure, radioactive surfaces, rail wheels.

The laser ultrasonic excitation can be performed by using pulse lasers, continuous lasers, or laser interferometry. The non-contact method has been preferably used in crack/corrosion detection, quantification, and localization of structural defects in various areas [65,110,111]. It can provide high spatial resolution hence damage diagnosis can be achieved more easily. Further, non-contact techniques are reported to be economical, easily applicable to harsh environments with little maintenance. The laser-based ultrasonic propagation imager (UPI) with piezoelectric sensors have been used for signal excitation and reception by Park and Lee [112] during inspection of submerged metallic structures, with a 2-mm crack. The employed method was successful in quantifying the defects on fully submerged surfaces in stationary/oscillating waters and non-submerged structures. It has been revealed that the measured crack size for both submerged cases increased slightly than actual crack size and the crack locations were observed to be slightly tilted due to refraction/change in impedance. The presence of liquid on one or both sides of the steel plate was investigated by Wilcox et al. [113] using EMAT in which slight attenuation (1 dB/m) of the S_0_ wave mode was observed, whereas the attenuation of A_0_ mode found to be around 57 dB/m. In a similar research, An et al. [109] used laser ultrasonic scanning system and a novel image processing technique to visualize crack on aluminium plates and found it a reliable defect characterization method.

**Figure 9:**
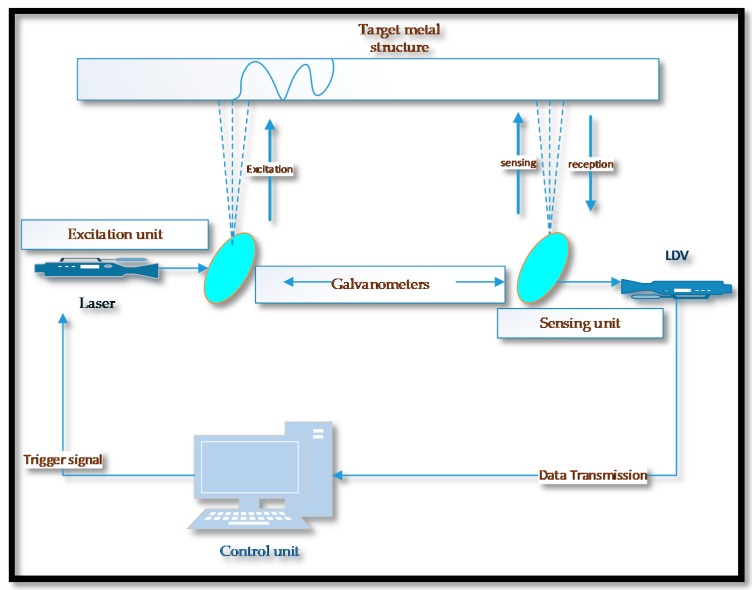
A fully non-contact laser ultrasonic scanning system.

Rao et al. [65] reported that the flaw detection in submerged structures using UGW is rather a more challenging because of the leaking wave energy into the liquid. In an experiment based research study on corrosion monitoring and prediction of wall thickness loss, Fromme [114] used transducer based GWs excitation and output signal response was measured through a laser interferometer. A good agreement between practical and theoretical predictions was achieved. The crack detection in submerged structure with a fully non-contact method has been reported by Lee et al. [111] in which image blurriness was found to be more dominant factor, in submerged condition. A schematic diagram of a complete non-contact laser ultrasonic scanning system (excitation and sensing) is illustrated in Figure 9.

The pulse-echo technique was used by Djili et al. [115] for detection of artificially crafted notches on submerged copper tubes. A single transducer was used for excitation and reception of leaky GWs and notches of various small sizes were detected with a greater accuracy. The response of GWs in aluminium-based aero structures was studied by Zhao et al. [4] and several complications were observed in wave propagation over a longer distance due to strong attenuation from surface paint and riveting. A novel algorithm and correlation analysis technique called Reconstruction Algorithm for Probabilistic Inspection of Defects (RAPID) was adopted for collection of output signal from rivet cracks and corrosion patches. This technique was found efficient in detection, size estimation and localization of defects.

A predictive numerical based study was conducted in a steel plate using acoustic emission (AE) method. It was revealed that peak amplitudes of antisymmetric (A_0_) signal is greater than that of symmetric (S_0_) signal [116]. They also observed that the peak amplitude of bulk wave was found to be less significant than that of the GWs peak amplitudes. Because of some special characteristics of A_0_ mode of GWs (i.e., out-off-the-plate displacements) they have been used as a preventive technique for bio-fouling on ship structures [44]. Carvalho et al. [117] used the ultrasounic automated system to inspect corrosion induced superficial defects in marine/ship structures. The artificial neural networks (ANN) has been used by some researchers as a reliable method in automatic ultrasonic testing and defect detection in pipelines [118]. 

Recently, Martinez et al. [119] presented a numerical modelling approach to study the combined effect of geometry complexity and residual stresses produced during the fatigue crack growth (FCG), using the lamb waves. Successful interaction of GWs with a crack size of 2.1 mm on aluminium structure has been reported. The drop in amplitude (due to attenuation) was recorded to be more significant in A_0_ wave mode than the S_0_ mode on the higher frequency. Fromme and Sayir [91] observed the scattering effect of A_0_ wave mode around rivet holes, notch cut and fatigue cracks in an aluminium plate. The piezo transducers were used for excitation of antisymmetric Lamb GWs and its scatter measure was taken with the help of laser interferometer. The field results depicted significant changes with the presence of a notch or a crack, which were much smaller than the wavelength of excited GWs.

In addition to the uses of Lamb guided waves, the review of the literature revealed that the S_H_ and Rayleigh waves are also used extensively in structural diagnostic studies of plate type and tubular shaped metallic/composite structures [34]. As during propagation in an elastic medium, S_H_ wave is polarized parallel to a surface, so they reflect without mode conversion. Additionally, during this process the amplitude changes are insignificant, therefore, S_H_ wave is considered to be superlative for crack detection in welds [35,120,121,122]. It has distinct capability of travelling across bended surfaces with minimal energy losses [55]. The S_H_ wave with EMAT was found effective in defect identification in a 7 mm thick carbon steel sample. It was found to be highly reliable in defect detection, axial sizing and approximation of circumferential positioning of a defect for both artificial cracks and corrosion defect, even with a thick paint coating [54]. The reduction of wall thickness of corroded pipelines has been quantified by Andruschak et al. [123] using S_H_ waves. The defect location and quantification for corrosion loss was clearly established from high amplitude reduction and delayed arrival timings of received signal.

**Figure 10:**
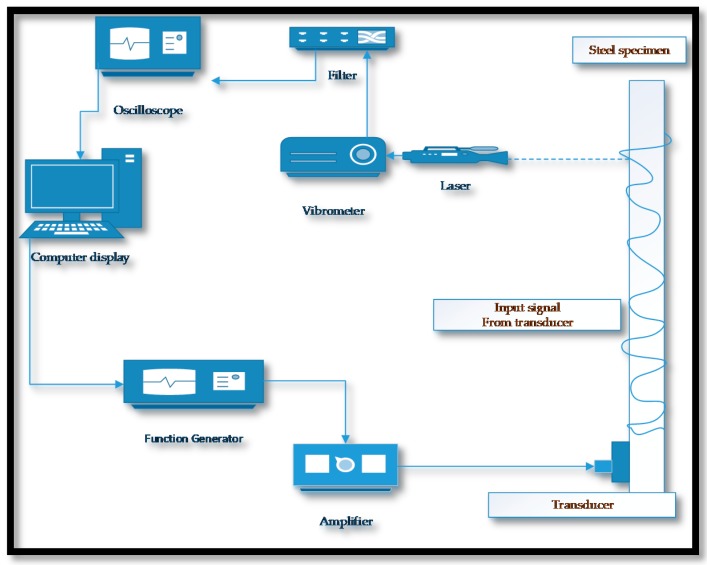
Experimental setup for laser measurement of UGWs on steel specimen using a partial non-contact method.

In ships and offshore marine platforms, accelerated rates of corrosion based deterioration has been reported, primarily due to the harsh seawater climatic conditions. The general corrosion and pitting occur more frequently. Apart from these, crevice, galvanic, fretting, groove, fatigue, edge and stress corrosion are also encountered in various loaded and dynamic loaded structures [124,125,126]. Fromme et al. [127] studied the corrosion based thickness loss of marine steel plates, partly immersed in salt water. A partially noncontact method was used in which the Lamb waves (A_0_ and S_0_) were simultaneously excited using wedge transducer (at a center frequency of 450 KHz) and the reflected signal was collected with a laser interferometer. The change in the response signal due to the wave mode interference was measured and the wall thinning was monitored. A good agreement was found between experimental results and theoretical estimation. In partial non-contact method, the contact less mechanism can be used for either excitation or sensing. This experimental setup is shown in Figure 10. The SHM methods face various impediments to distinguish the variations due to structural defects and those occur by transitions in environmental and operational conditions. The active SHM method of UGWs is built on the principle of the difference between the most recent time-trace (with damage) and a baseline time-trace recorded in absence of structural damage. The residual signal after the subtraction of a baseline time-trace is referred to as a remaining time-trace which can be effected by the significant changes in environmental factors, particularly the temperature, and surface conditions [128,129]. These variations may adversely affect the efficiency of the subtraction process and the residual time-trace may be amalgamated with the coherent noise signal because of the shift in arrival timing of wave-packets from structural topographies. This degradation occurs due to the change in the velocity of acoustic waves and thermal expansion of structure [9]. In order to minimize the effect of temperature variations on the performance of GWUT, some researchers have proposed compensatory strategies such as optimal baseline selection (OBS), baseline signal stretch (BSS) and a combination of both [9,129,130,131]. In [9], the authors recommended that these compensatory techniques are also valid for numerous other climatic variations, provided they tend to result in a homogenous and isotropic change in the velocity of the guided wave.

Some researchers have reported that the minor temperature change (up to 40 °C) does not affect the performance of GWs therefore special compensation techniques may not be required, except in cases where the structural damage to be sensed is very small in size [40]. The research work of Croxford et al. [132] revealed that signal fluctuations even due to minor changes in temperature is enough to generate significant coherent noise (−25 dB), rendering the use of compensatory measures. The variation in TOF of guided waves with the increase in temperatures (20–150 °C) has been monitored in [133]. A small error in location of defect was recorded till 80 °C, however, a significant decrease in sensitivity was recorded beyond this temperature.

**Figure 11:**
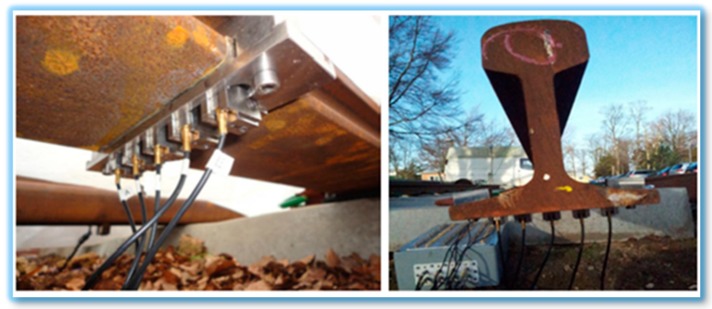
GWUT transducer mounting for rail foot inspections (adapted from [52]).

During a research study on SHM of rail tracks, Moustakidis et al. [52] presented that the variation in ambient temperature and humidity can mask changes in the output signal due to structural damage. It was also noticed that certain factors such as rain and high temperature play a significant role in deteriorating the damage detection capability of GWs used with contact method. Figure 11 shows the experimental setup of this research.

Performance of transducers is crucial in the GWUT especially under variant operating conditions. This performance is highly influenced by several external factors such as environmental dynamics, high operating temperatures in particular. As the general purpose piezoelectric sensors used in UGWS have low curie temperatures, therefore their signal reception performance deteriorates significantly in higher temperatures. The intrinsic E/M impedance, admittance signatures of PWAS degrades when exposed to higher temperatures [134]. In these operating conditions, designed piezoelectric transducers (lithium niobate) are preferred, especially in high temperature operating conditions such as nuclear reactors, boilers and gas turbines [72]. 

**Figure 12:**
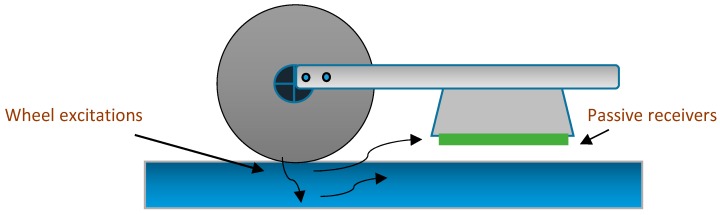
The passive inspection prototype for the field tests using non-contact air receivers.

In some cases, non-contact GWs methods have also been preferred for SHM and NDE at elevated temperatures [108]. Several researchers have established the passive SHM concept on rail tracks, where the dynamic excitation signature is triggered by the external source (i.e., rolling wheels of a train) and non-contact air-coupled ultrasonic receivers may be placed underneath a testing railcar for rail examination. An experimental study to assess the performance of the passive ultrasonic inspection of rail has been demonstrated in [135], at train speeds up to 80 mph. The problems such as highly variable energy excitation from rotating train wheels were resolved using the deconvolution operator and Green’s function. Upon successful completion of field tests, this innovative concept is expected to perform efficient health inspections of rail over longer lengths, with a minimal or no disruption of regular traffic. A passive inspection mechanism is illustrated in Figure 12.

## 4. Discussion and Proposed Methodology for Future Research

The role of health monitoring in operational availability, upkeep, and maintenance of equipment and structures has increased exponentially in recent years. Conventional maintenance procedures such as corrective and preventive are transforming rapidly towards technology-driven upkeep methods (such as CBM, SHM, etc.) due to an ever-increasing reliance on progressive technological tools. The role of these technologies is more vital in equipment health prognostic studies, RUL assessment and through-life engineering support (TES) of structures, especially in cases when they are operating away from base maintenance facilities, e.g., aircraft, ships, subsea pipelines, and offshore energy structures. 

The GWUT is a powerful and rapidly evolving technology with enormous potentials to be used as NDT and SHM of various platforms. Its long-range defect diagnostic capability with minimal preparatory costs (i.e., without paint, insulation and lagging removal) for inspection is the biggest competitive edge over contemporary diagnostic techniques. Contrary to the limitations of other types of defect diagnostic technologies, guided waves have a lower level of operational barriers for usage in any type of material and environmental conditions. They have been used enormously in magnetic/non-magnetic, metals/composites isotropic/anisotropic structures of various shapes. The flexibility of use with contact or non-contact methods has enhanced its viability and future scope. The latter is relatively a more sophisticated technique, which has frequently been used in recent days for investigation of defects in complex, sensitive, dynamically loaded structures, especially in high temperatures where the use of simple contact type sensors may not be appropriate for use. Nevertheless, the contact transducer technique is still the most widely used method in UGWs because of its low initial cost, the flexibility of use, and portability.

In the reviewed literature, the efficacy of GWUT in different materials, operating and environmental conditions has been studied through experimental and numerical methods. This technique has been successfully used to locate and quantify the defects in various types of structures by classification of variations in numerous parameters of output signals, like frequency, amplitude, reflected/transmitted coefficient, velocity, TOF, and impedance. Certain limitations of UGWs have also been deliberated in the literature especially in bends/joints, complex geometries, submerge and thickly painted structures. These performance degradations have been attributed to the highly dispersion nature, scattering effects and attenuation of some acoustic guided waves under specific conditions. In the real world situations, the performance of UGWs can be affected by intermingling/interference of external acoustic signatures of running machinery (in surrounding) with the of GWs signals. More improvements in the use of GWs methodology is still needed to overcome the highlighted impediments, increase reliability, effectiveness, and diagnostic ability for identification of various defect features. 

The Lamb wave interaction with damaged surface influences their propagation characteristics significantly, tempting various distinctive scattering phenomena (transmission, reflection, mode conversion); dependent on locations and severity of damaged area. The omnidirectional scattering (around the hole) of GWs has been reported in literature, when interacted with a through-thickness hole in the composite surface. The positioning of the sensor with respect to crack location becomes crucial in cases where the cracks are oriented in specific dimension/directions, shapes and angles. Therefore, the development of meticulous damage identification approach with an appropriate number of sensors would be required to capture the scattered waves optimally.

The literature has reported that the angle of crack orientation and length of crack has an important relationship with the reflection and transmission coefficients of incident elastic waves in plate-like metal surface. It has been revealed by some researchers that the reflection coefficient decreased whereas the transmission coefficient increased with the crack angle. This relationship was found to be even more complex in case of change of length of crack and angle. However, it is suggested that the reflection coefficient alone may not be able to define the length of the crack/notch exclusively. Moreover, the notch width can also influence the reflection and transmission ratio of GWs. Similarly, some research studies have reported that the transmission coefficient is higher in half through-thickness crack than in the full through-thickness, because of the fact that the GWs travel in the remaining half through-thickness part of the metallic surface.

The estimation of a crack depth has a great significance in determining the severity of crack and pit to crack transition. In absence of near-surface boundaries in metallic plates, the reduction in transmitted signal between actuator and sensor can be attributed to the quantitative measure of the defect. Nevertheless, in real-world situations the performance of active SHM with GWs can be adversely influenced by external excitation signatures; hence, defect investigation process can be further complicated. In the applications where external excitations are significant, passive SHM methods (such as acoustic emission) have been used by some researchers with contact/contactless methods. For example, the contact between wheels of train and rail track generates high excitation acoustic emissions which have been used for health inspection of rail tracks using non-contact methods.

Marine structures are often subjected to corrosive seawater conditions and enormous stress levels due to internal and external loading conditions. Therefore, the rate of various degradation mechanisms and failure probability are quite phenomenal. In research literature, the crack identification on water submerged metal structures has been deliberated at length. It has been reported that the defects were detected successfully with minor variations in the location of actual defects. This variation may be accredited to refraction phenomena in the denser medium. Behaviour of various modes of guided waves have been investigated to control antifouling process, detection of ice layers and de-icing in the ship’s underwater hull, aerospace, and wind turbine structures. Many experiment/numerical based research studies have presented that the GWs can be used effectively to differentiate between the ice layer, water content and structural defects on external surfaces of engineering structures that are operating under dynamic climatic conditions.

The GWUT can be extremely useful for SHM, fracture diagnostics in obscure locations and subsequent CBM planning of dynamically loaded marine structures. It has been reported that presently the structural integrity inspection of ships and associated harbour installations (floating docks) is carried out using either conventional NDT methods like bulk ultrasonic waves, which require complete removal of surface protective paints or destructive methods like sand or grit blasting. These inspections can only be performed in dedicated graving/floating docks, which make inspection tasks expensive, labour intensive and time-consuming. The use of autonomous vehicles to facilitate video imaging of underwater structures and inspection of difficult to access areas (to identify the severity of corrosion, damage and anode wear) has already been reported in the literature [136,137,138]. A comparatively new method with integration of GWUT (contact/contactless methods) and remotely operated vehicles (ROV) or autonomous underwater vehicles (AUV) can be introduced as future health inspection/prognostic technique for ships and other marine structures. 

The literature review disclosed that the decision-making for the sensor(s) placement methods (pulse-echo, pulse-catch and a number of sensors) is extremely important. The precise identification/quantification of defect features (e.g., orientation, size and relative position of cracks) is very much reliant on the selection of crack detection approach and the positioning of the sensors. The experimental results on metallic and composite plates presented in [40,95] would be instrumental to develop a relationship between the directionality of defect features (in-line or offset position with respect to the direction of incident GWs), and the reflection/transmission ratio of induced guided waves. Although, the variation in the reflection/transmission coefficient with the orientation of defect in a particular direction has been elaborated. However, the aspects of identification/quantification of defect orientation and shape have not been discussed, probably because of nonlinearity/uncertainty of scattering data with the variation in the length of notch/crack. Therefore further research work can be conducted to explore a more realistic relationships and methodologies.

Various breakthroughs have been reported on defect characterization in metallic structures. However, to the best of knowledge of the authors of this paper, the research publications for determination of shape and orientation of structural defects in plate type metal structures are still obscure. The prominence of these two parameters (shape and orientation) is extremely vital in the purview of fatigue and fracture mechanisms, especially for the structures working under extreme static/dynamic loading conditions. The fatigue growth propagation and pit to crack transition processes are highly dependent on shape and orientation of the developed cracks in metallic structures, specifically those operating under complex loading and corrosive conditions. The identification of these prominent features of structural defects can be even more interesting than just detection or location of the defect. Advanced working methodologies, statistical/optimization tools and experimental equipment would be desirable for interpretation and empirical formulation of output (reflected/transmitted) signals. Therefore, the use of advanced and high-resolution equipment (Lecory wave-runner oscilloscope-Xi series), actuators/sensors (PAC’s integral preamp- WDI-AMP) would be instrumental to minimize the equipment driven uncertainties.

**Figure 13:**
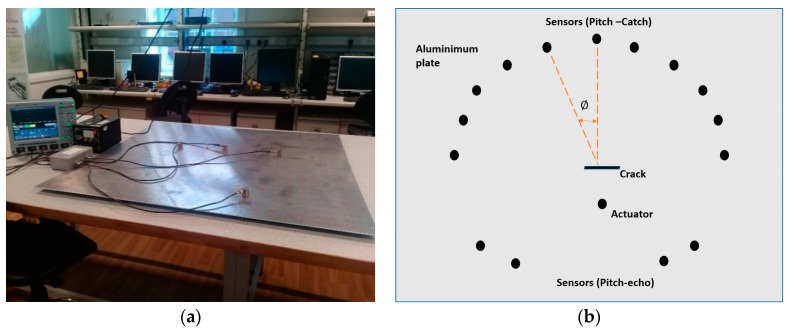
Proposed experimental set-up (**a**) and methodology (**b**) for placement of piezoelectric transducers.

This paper proposed an experimental-based research study to determine the shape and orientation of structural defect. The cracks/notches in several shapes and orientations can be artificially crafted on thin plate type aluminum plates. Formulation of an empirical model is possible based on variations in output signal parameters on various sensors placed in different configurations, as illustrated in Figure 13. From the literature review, it is asserted that the reflection and transmission coefficient varies nonlinearly with the change in orientation, shape and size of the structural damage. Therefore, the proposed sensor arrangement (in Figure 13b) will be helpful to detect the optimized reflected/transmitted coefficient patterns, with variation of defect features. Similarly, the change in the actuator position with respect to the crack would also affect the scattering pattern of GWs. This information (identification of scattering pattern vis-a-vis defect characteristics) is extremely beneficial in development of the empirical model. The acquired experimental results can be validated numerically by simulation software such as COMSOL Multiphysics or ABAQUS. 

## 5. Conclusions and Future Works

The UGWs have immense potential in various types of structures for CM, SHM, and NDE. They have the propensity to be used without disrupting the ongoing operations in the structure under investigation, be it a pipeline embedded in soil/insulation applied exhaust/air conditioning ducts, rail track, aircraft, or a marine structure. The use of various modes of guided waves have been reported for ice detection, de-icing and differentiation between actual defect, ice layer and pure liquid phases in structural applications. 

The flexibility of using GWs technology with several methods (contact, non-contact, semi contact and passive SHM) provide greater opportunities to extend its scope and applications in various other fields. To overcome the limitation of GWs in variant environments, various methodologies (OBS, BSS) have been adopted as compensatory strategies to optimize the performance. The potential of Lamb guided wave modes has been accepted in health monitoring of marine structures, aircraft, wind turbine blades, pigging of pipelines and antifouling on ship structures. Researchers have recognized the potential of UGWs to localized defects in various geometries and complex structures; hence, it provides the opportunity to analyze the severity of the defect, optimize inspection/maintenance interval and predict failure probability and RUL of valuable assets. 

During this review, it was established that no research work has been published yet suggesting a methodology or an empirical method to determine the shape, widths, depths, and orientation of defects in thin plate structures. In the proposed research methodology, cracks/notches on thin metal plates will be produced artificially in various geometries (shapes and orientation). In the proposed method, the defects may be investigated at various angles with respect to the position of transducers. Upon successful identification of defect geometries, an empirical model would be designed to predict the defect features and quantification. The numerical study can be conducted for validation and optimization of designed experiment and its methodology.

The outcome of experimental results based on this proposed plan can be of highly valuable to bring further improvement in GWUT based SHM/NDE in various field applications, particularly for identification of damage tolerance of structural defects and subsequent prediction of RUL and life extension (LE). It can be made as a part of future structural maintenance/inspection strategy of offshore installations and ships, which normally operates with limited man-power and away from base maintenance facilities. The big-data of SHM acquired from large marine structures (using ROV/AUV integrated GWs methods) can be continuously provided to the remote maintenance experts, through wireless transmission or internet of things (IOT). In presence of ground maintenance teams, this data can be interpreted and the defect (if any) can be investigated through comparison of data (existing versus historical SHM data). Subsequently, this priori information on structural health can be used to update/optimize the forthcoming maintenance/inspection schedules, using probabilistic inference methods such as Bayesian inference. Determination of characteristics and directional properties of defects (shape, depth, orientation, the relative position of crack) through GW technologies can be extremely helpful to predict the future growth rate and defect intensity. Notwithstanding, further research studies are essential to minimize the glitches with the use of guided wave technology for health monitoring of assets in practical field as well as to increase its reliability and effectiveness, under vibrant operating conditions 

Although some researchers have developed the experimental-based compensatory methods to uplift the performance of GWUT affected by the environmental (mostly temperature) variations. However, implementation of these benign methods in the field experiments is not free from numerous complications due to the abrupt transient behavior of a wide range of climatic factors which ca be triggered simultaneously. The ship’s hull is consistently under static/dynamic loading conditions from internally (machinery) or external factors (momentum/wave action) which aggravate the challenges for effective SHM, exponentially. Contrary to the lab experiments, the GWUT based health inspection of these giant structures involves several factors at the same time, which have the tendency to adversely affect the defect diagnostic capability. A few prominent factors are the non-uniform thermal gradients, wetting/icing of external surfaces, exterior acoustic signatures, and the surface protective measures. In the future research studies, field-oriented experimentation under marine weather conditions is necessitated to explore the capability of various GWs (Shear, Lamb, etc.) on large and valuable marine assets. The FEM approaches can also be used for the validation process, which is a cost-effectiveness way to evaluate the proficiency of GWs for characterization of salient defect features.
